# Color Adjustment and Fluorescence Matching of Single-Shade Composites: Spectrophotometric and Photographic Analysis Techniques

**DOI:** 10.3390/polym18151819

**Published:** 2026-07-25

**Authors:** Buket Karalar, Merve Nur Yılmaz, Merve Pelin Dur Sarıgüzel

**Affiliations:** 1 Department of Restorative Dentistry, Faculty of Dentistry, Sakarya University, 54187 Sakarya, Turkey; mpelindur@sakarya.edu.tr; 2 Department of Restorative Dentistry, Faculty of Dentistry, Bolu Abant İzzet Baysal University, 14030 Bolu, Turkey; mervenur.yilmaz@ibu.edu.tr

**Keywords:** color adjustment, cross-polarized photography, esthetic dentistry, fluorescence, single-shade composite, spectrophotometer

## Abstract

Background: Although the color adjustment of single-shade composites has been widely investigated, limited information is available regarding their fluorescence-matching performance. The aim of this study was to compare the optical performance of different composite resin systems by evaluating color adjustment under visible-light conditions and fluorescence matching under ultraviolet (UV) illumination. Methods: Standardized cervical cavities were prepared in 45 extracted human maxillary central incisors. Teeth were divided into nine groups (*n* = 5): five single-shade composites (Omnichroma, Charisma Topaz One, Vittra APS Unique, Zenchroma, and ONEshade), two simplified shade composites (Optishade and Clearfil Majesty ES-2), one multi-shade composite (Estelite Sigma Quick), and one group-shade composite (G-aenial A’chord). Color adjustment was evaluated using spectrophotometry (SP) and cross-polarized digital photography (CP), whereas fluorescence matching was evaluated using fluorescence-illuminated digital photography (FI). Data were analyzed using generalized estimating equations and Kruskal–Wallis tests. Results: FI showed significantly higher ΔE values (23.01 ± 7.04) than SP (3.34 ± 1.52) and CP (3.55 ± 1.35) (*p* < 0.001). G-aenial A’chord exhibited the lowest ΔE values (6.54 ± 5.01), whereas Charisma Topaz One showed the highest fluorescence mismatch (30.22 ± 10.92) (*p* < 0.001). A significant interaction was found between composite type and measurement method (*p* < 0.001). Conclusions: While single-shade composites exhibited favorable color adjustment under visible-light conditions, they showed greater fluorescence mismatch under UV illumination. In contrast, the group-shade composite demonstrated the most stable optical performance across different evaluation methods.

## 1. Introduction

In addition to composite resins that are available in multiple shades with varying opacities and color characteristics, single-shade composite resins have been developed in recent years to simplify clinical procedures. These materials can adapt to natural tooth structures across a wide range of shades despite being available in only one shade [1,2,3]. This adaptation is based on an optical phenomenon known as the “chameleon effect,” which allows a composite resin to visually blend with and adapt to the color of the surrounding tooth structure [2]. This effect is associated with the scattering, absorption, and reflection of light within the material and is influenced by physical properties such as translucency, refractive index, and filler particle structure [1].

The ability of restorative materials to match the appearance of natural tooth structure is critical to the success of esthetic restorations. Accordingly, color adjustment can be evaluated using both objective and subjective methods. Spectrophotometry (SP) is considered one of the primary objective evaluation methods [4]. Spectrophotometric measurements are regarded as the gold standard for color analysis because they are minimally affected by environmental conditions and provide high reproducibility [5,6].

Among the subjective and semi-objective evaluation methods, digital photography using cross-polarization filters has emerged as a key technique. Cross-polarization eliminates specular surface reflections, allowing more accurate visualization of the true color of the material. This method reduces errors caused by glare, misinterpretation of opacity differences, and light reflections, thereby enabling a more reliable evaluation of color matching between the restoration and the tooth structure. Consequently, the optical adaptation of composite resins in clinical settings can be analyzed more objectively using digital imaging [7].

Another important optical property that contributes to the natural appearance of esthetic restorations is fluorescence [8]. Natural tooth structure exhibits characteristic blue-white fluorescence under ultraviolet (UV) illumination [9]. Therefore, restorative materials with fluorescence properties similar to those of natural teeth can improve the harmony between restorations and surrounding dental tissues, particularly under different lighting conditions [10]. Fluorescence matching helps reproduce the optical appearance of natural tooth structure under UV illumination and may complement conventional color assessment across different lighting environments [11].

In addition, fluorescence-based sensors and biofluorescence imaging have emerged as valuable diagnostic tools in dentistry given their ability to detect changes in dental tissues, bacterial by-products, and dental deposits with high sensitivity, thereby expanding the clinical applications of fluorescence beyond esthetic assessment [12,13]. These applications further underscore the importance of accurately reproducing the fluorescence characteristics of restorative materials to better mimic natural tooth tissues under various illumination conditions [12].

Despite the growing applications of fluorescence in dentistry, studies examining the fluorescence-matching performance of restorative composite resins remain limited. Although several studies have investigated the fluorescence properties of composite resins, only one study has specifically focused on single-shade materials [14], indicating that evidence on the fluorescence-matching behavior of contemporary single-shade composites remains limited. Moreover, the evaluation of only two single-shade universal composites in that study limits a comprehensive understanding of the optical behavior of these materials. Therefore, the present study comparatively evaluated five single-shade and two simplified-shade composite systems alongside group-shade and multi-shade control groups. In addition, the inclusion of a broad range of contemporary composite systems and the integration of multiple evaluation methods provide a comprehensive comparative assessment of the color adjustment performance and fluorescence matching of single-shade composites, thereby enhancing the study’s originality and clinical relevance.

The aim of this study was to evaluate the optical performance of different composite resin systems under both visible light and fluorescence conditions by assessing color matching using spectrophotometry (SP) and cross-polarized photography (CP) and fluorescence matching using fluorescence-illuminated photography (FI). Specifically, the study aimed to compare the color- and fluorescence-matching performance of contemporary single-shade and conventional composite resin systems using complementary instrumental and photographic evaluation methods and to determine whether restorative materials that exhibit satisfactory optical integration under conventional lighting conditions maintain their optical harmony under fluorescence illumination. Accordingly, three null hypotheses (H_0_) were established: (I) the measurement method (SP, CP, and FI) does not significantly affect the evaluated optical outcomes; (II) the composite resin type does not significantly affect the evaluated optical outcomes; and (III) no significant interaction exists between measurement method and composite resin type.

## 2. Materials and Methods

### 2.1. Sample Size Analysis

The sample size was calculated using G*Power 3.1.9.4. Based on a significance level of 0.05, a power of 95%, and an effect size (f = 1.0968) derived from a previous study [14], the minimum required sample size was 4 specimens per group. To compensate for potential specimen loss and improve statistical reliability, five specimens were included in each group, resulting in a total sample size of 45.

### 2.2. Tooth Selection and Restoration Protocol

For the study, a larger pool of human maxillary right and left central incisors extracted for periodontal reasons was collected and stored in 0.1% thymol solution. Teeth with prior restorations, carious lesions, or hypomineralization were excluded. The shades of teeth were evaluated using a spectrophotometer (Rayplicker, Borea, Limoges, France), and only those with an A2 shade were included in the study (*n* = 45).

The selected specimens were randomly allocated to nine experimental groups (*n* = 5) based on different composite resins, including five single-shade composites [Omnichroma (OM), Charisma Topaz One (CTO), Vittra APS Unique (VAU), Zenchroma (ZC), and ONEshade (OS)], two simplified-shade composites [Optishade (OPS) and Clearfil Majesty ES-2 (CME)], one multi-shade composite [Estelite Sigma Quick (ESQ)], and one group-shade composite [G-aenial A’chord (GAC)]. Detailed information on the composite resins used in the study is presented in Table 1.

Each tooth was subjected to a single standardized Class V restoration using only one composite material. The same restored tooth was subsequently evaluated using all three measurement methods (SP, CP, and FI).

Cavity preparations were performed under continuous water cooling with green-banded round diamond burs, which were replaced after each procedure. Cavity dimensions were standardized to 2 mm in the inciso-gingival direction, 3 mm in the mesiodistal direction, and 2 mm in depth. A 45° bevel was prepared along the sharp enamel margins. After cavity preparation, the cavity surfaces were etched with 37% orthophosphoric acid (Ultradent Etchant, Ultradent Products, South Jordan, UT, USA) according to the manufacturer’s instructions. A universal adhesive system (Clearfil Universal Bond Quick; Kuraray Noritake Dental, Okayama, Japan) was applied and polymerized for 10 s using an LED light-curing unit (ZenoLite, President Dental GmbH, Allershausen, Germany) at an irradiance of 1200 mW/cm^2^. The output intensity of the light-curing unit was verified with a radiometer (Bluephase Meter II, Ivoclar Vivadent AG, Schaan, Liechtenstein) before each use.

The composite resins were placed in the cavities in layers, and each layer was polymerized according to the manufacturer’s instructions. Low-speed, two-step diamond-impregnated spiral polishers (Hager & Meisinger, Neuss, Germany) were used for finishing and polishing. The specimens were stored in distilled water at 37 °C for 24 h before evaluation.

After 24 h of storage, the specimens were removed from distilled water, gently blotted dry with absorbent paper to remove excess surface moisture, and immediately analyzed by SP, CP, and FI. All measurements were performed under standardized hydration conditions.

### 2.3. Evaluation Protocol

All spectrophotometric color measurements were performed using the same spectrophotometer. The device was calibrated before each measurement according to the manufacturer’s instructions, and all measurements were performed on a neutral white background under standardized D65 illumination. L*, a*, and b* color coordinates were obtained from both the restorations and the adjacent natural tooth structure. Three consecutive measurements were recorded for each specimen, and the mean L*, a*, and b* values were calculated. The L* coordinate represents lightness (0–100), whereas the a* and b* coordinates represent the red–green and blue–yellow chromatic axes, respectively. The mean color coordinates were then used to calculate the CIEDE2000 color difference (ΔE_00_), which was used to assess the color adjustment level of the specimens [7].

$${\Delta E}_{00}={\left({\left(\frac{\Delta L}{K_{L}S_{L}}\right)}^{2}+{\left(\frac{\Delta C}{K_{C}S_{C}}\right)}^{2}+{\left(\frac{\Delta H}{K_{H}S_{H}}\right)}^{2}+R_{T}\left(\frac{\Delta C}{K_{C}S_{C}}\right)\left(\frac{\Delta H}{K_{H}S_{H}}\right)\right)}^{1/2}$$

where ΔL′, ΔC′, and ΔH′ are the differences in lightness, chroma, and hue, respectively, between the two samples being compared. The weighting functions for the lightness, chroma, and hue components were SL, SC, and SH, respectively, and KL, KC, and KH were parametric factors adjusted based on different viewing parameters; however, in this study, they were set to 1. The perceptibility (PT) and acceptability (AT) thresholds were set at 0.8 and 1.8 units, respectively [5].

**Figure 1:**
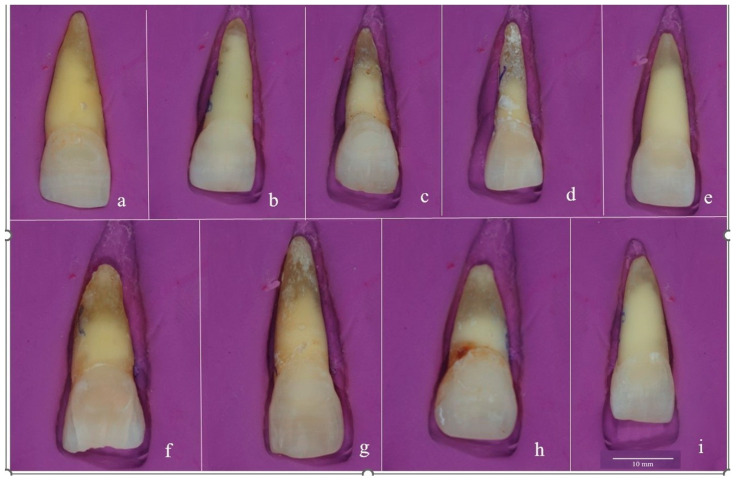
Cross-polarized photographic analysis of composite brands: (**a**) Omnichroma, (**b**) Charisma Topaz One, (**c**) Vittra APS Unique, (**d**) Zenchroma, (**e**) ONEshade, (**f**) Optishade, (**g**) Clearfil Majesty ES-2, (**h**) Estelite Sigma Quick, and (**i**) G-aenial A’chord.

**Figure 2:**
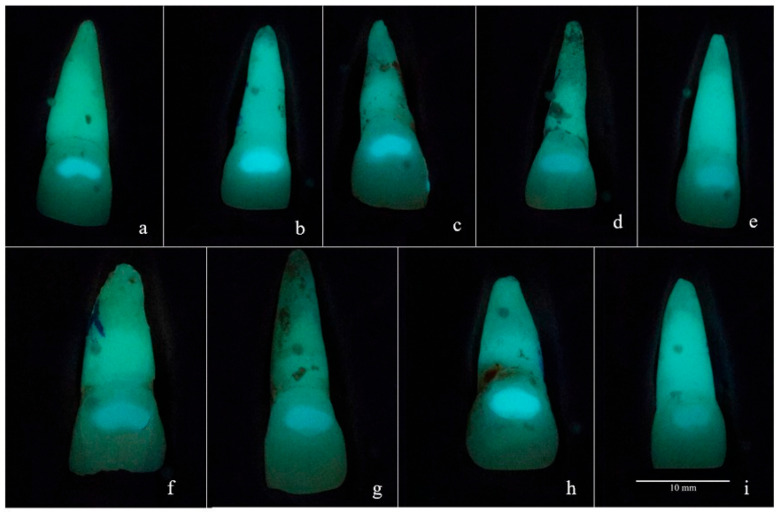
Fluorescence-illuminated photographic analysis for composite brands: (**a**) Omnichroma, (**b**) Charisma Topaz One, (**c**) Vittra APS Unique, (**d**) Zenchroma, (**e**) ONEshade, (**f**) Optishade, (**g**) Clearfil Majesty ES-2, (**h**) Estelite Sigma Quick, and (**i**) G-aenial A’chord.

CP and FI images were obtained for each restoration. The imaging system consisted of a Canon EOS R7 DSLR camera (Canon Inc., Tokyo, Japan) equipped with a 100 mm macro lens (Canon RF, Tokyo, Japan). CP photography was performed using a dual-flash system (24EX, Canon, Tokyo, Japan) and cross-polarization filters (Cross-Polar, Vilnius, Lithuania). Camera settings were standardized at f/22, ISO 400, and 1/125 s (Figure 1). FI photography was performed using a black PhotoBox (PULUZ, Shenzhen, China) and a dual-fluorescence light source (ASHUR, Hangzhou, China; 365 nm wavelength, 218 mW radiant power) (Figure 2). For FI imaging, the camera settings were set to f/22, ISO 8000, and 1/125 s.

The L*, a*, and b* values obtained from the images were analyzed using the “Info” panel of Adobe Photoshop CS6 (Adobe Systems Inc., San Jose, CA, USA) [8]. To increase measurement reliability, three repeated measurements were obtained from each region. The ΔE_CP_ and ΔE_FI_ values were calculated using the CIEDE2000 formula. In addition, b* values obtained from the RGB (red, green, and blue) color space were used to evaluate fluorescence matching.

### 2.4. Statistical Analysis

Statistical analyses were performed using IBM SPSS Statistics version 31 (IBM Corp., Armonk, NY, USA). The normality of the data distribution was evaluated using the Shapiro–Wilk test. Data were analyzed using the generalized estimating equations (GEEs) model for repeated measurements, whereas the Kruskal–Wallis test, followed by a Bonferroni-corrected Dunn post hoc test, was used for non-normally distributed data. The level of statistical significance was set at *p* < 0.05.

## 3. Results

According to the generalized estimating equation (GEE) analysis presented in Table 2, ΔE values differed significantly according to the composite type (*p* < 0.001). Regardless of the evaluation method, GAC exhibited significantly lower ΔE values than the other composite groups (6.54 ± 5.01; *p* < 0.001). The ΔE values of the remaining composites ranged from 9.08 to 12.73, with no significant differences (*p* > 0.05). Detailed pairwise comparisons for the composite main effect are provided in Table S1.

When evaluated using the measurement methods, significant differences were observed in ΔE values (*p* < 0.001). The overall mean ΔE values were 3.34 ± 1.52 for SP, 3.55 ± 1.35 for CP, and 23.01 ± 7.04 for FI. According to the letter-grouping analysis, no significant difference was found between the SP and CP methods, whereas the FI method showed significantly higher ΔE values. Estimated means and pairwise comparisons for the measurement method are presented in Table S2.

Furthermore, a significant interaction was observed between the composite type × evaluation method for ΔE values (*p* < 0.001). The lowest ΔE value was observed in the GAC-SP group (2.61 ± 1.36), whereas the CP value for the same composite (3.90 ± 1.47) was significantly higher. In general, the FI method yielded higher ΔE values. The highest values were observed in the CTO-FI (30.22 ± 10.92) group, whereas the VAU-FI and ZC-FI groups (20.08 ± 5.62) showed intermediate values. Overall, ΔE values obtained with SP and CP methods were lower than those obtained with the FI method. Simple method effects within each composite material are provided in Table S3. 

As shown in Table 3, the median b*ΔE_FI_ values across composite types were significantly different (*p* < 0.001; η^2^ = 0.83, 95% CI [0.10–1.38]). The median values were 211 (200–223) for CTO, 145 (135–160) for CME, 214 (192–216) for ESQ, 133 (100–149) for GAC, 197 (194–206) for OC, 154 (143–161) for OS, 158 (156–162) for OPS, 179 (172–210) for VAU, and 154 (125–163) for ZC. Pairwise comparisons revealed significant differences between CTO and CME, GAC, and ZC; between CME and ESQ; between ESQ and GAC; and between GAC and OC (*p* < 0.05). No significant differences were observed for the remaining comparisons (*p* > 0.05).

## 4. Discussion

In this study, we comparatively evaluated the color adjustment and fluorescence-matching performance of contemporary composite resin systems using modern optical assessment methods. Spectrophotometry (SP) and cross-polarized photography (CP) were used to assess color adjustment under visible-light conditions, whereas fluorescence-illuminated photography (FI) was used to evaluate fluorescence matching under UV illumination. The findings demonstrated that both the measurement method and the composite resin type significantly influenced the measured optical properties. Consequently, all null hypotheses established in the present study were rejected.

Although color-matching technologies have been developed to improve the accuracy of shade selection, the subjective nature of visual methods and the lack of standardization among existing shade guides remain important limitations [15]. Therefore, spectrophotometers are frequently preferred because they provide more objective and reliable results [7]. In recent years, the CP method has emerged as an effective alternative because it eliminates specular reflections, provides more homogeneous illumination, and allows clearer evaluation of dentin and enamel characteristics [16,17]. Previous studies have reported that the CP method yields reliable results comparable to those obtained with the SP method [15,18,19]. Accordingly, the CP method was preferred in the present study as a practical option for clinical applications. In contrast, FI was not intended to assess color adjustment but to evaluate fluorescence matching, a distinct optical property that contributes to the esthetic appearance of restorative materials under fluorescence illumination. The findings were consistent with the literature, as no significant difference was observed between ΔE_SP_ and ΔE_CP_ values [15]. Despite its high accuracy and reliability in color analysis, SP has certain drawbacks such as high cost and limited availability in routine clinical settings. In such cases, the CP method may serve as a more accessible and practical alternative. Furthermore, the numerical data obtained from spectrophotometric measurements are often difficult for patients to interpret. In contrast, digital images acquired using the CP method can be shared with patients, potentially strengthening patient–clinician communication, increasing patient involvement in the treatment process, and improving patient satisfaction.

To clinically interpret the optical performance of restorative materials, it is essential to evaluate color differences according to PT and AT threshold values. In this context, the widely accepted 50:50 perceptibility (PT: 0.8) and acceptability (AT: 1.8) thresholds of the CIEDE2000 system reported in the literature were used as references to analyze the clinical relevance of the present findings [5,20]. Şahin et al. [21] found that cavity depth was a determinant of color matching in cervical restorations using the CP method. The authors demonstrated that values obtained at a 1 mm cavity depth remained within clinically acceptable limits (OM: 1.86; CDO: 1.45), whereas the AT threshold was exceeded at a depth of 2 mm. This phenomenon has been attributed to the reduction in background effects with an increase in material thickness, which weakens the color adjustment potential (CAP) mechanism [6]. Consistent with these findings, the present study also demonstrated that ΔE_SP_ and ΔE_CP_ values in 2 mm deep cavities exceeded the clinically acceptable threshold. However, given the time-dependent dynamics of CAP, the SP and CP findings obtained at the early stage may reflect the initial optical condition of the materials rather than their final color adaptation. Therefore, these relatively high values should not be interpreted as clinical failure but rather as early-stage responses of the materials before optical stabilization.

In the present study, only GAC showed statistically significant differences from the other groups in ΔE values obtained using the SP and CP methods. In the literature, Korkut et al. [22] reported that group-shade composites subjected to a shade-selection procedure exhibited superior color matching compared with single-shade composites in a study on acrylic teeth using the CP method. Similarly, in the study by Şahin et al. [21], two single-shade composites (OM and CDO) and one group-shade composite (NeoSpectra) were evaluated, and the best color match was observed in the group-shade composite. Consistent with these findings, Alhamdan et al. [23] and Fahim et al. [24] also reported that group-shade composites demonstrated better color matching than the single-shade OM composite. Taken together, these findings indicate that group-shade composites may exhibit more stable and predictable optical behavior.

In the present study, the fluorescence-matching behavior of single-shade composite resins was compared with that of multi-shade and group-shade composites using ΔE values calculated from digital images acquired under UV illumination. The UV wavelength (365 nm) and the standardized imaging protocol were selected in accordance with previously published fluorescence imaging methodologies. In addition, the use of a standardized PhotoBox system ensured consistent image acquisition conditions throughout the study [8,14,25,26].

Cruz et al. [11] comparatively evaluated the fluorescence properties of nine composite resin systems using spectrophotometry and digital photographic analysis, reporting that fluorescence varied by material type. Similar findings have been reported by Jablonski et al. [27] and Meller et al. [28], demonstrating that fluorescence is highly material-dependent. Consistent with these observations, the present study demonstrated a significant interaction between measurement method and composite type, indicating that optical outcomes were influenced not only by the intrinsic properties of the restorative materials but also by the evaluation method. This difference may be explained by methodological variations among the studies. While Cruz et al. [11] evaluated the intrinsic fluorescence of composite specimens directly, the present study assessed fluorescence matching between the restoration and the adjacent natural tooth structure using ΔE values obtained under UV illumination. The intrinsic fluorescence and optical characteristics of the natural tooth structure may alter light interaction with restorative materials, thereby contributing to the significant interaction observed between measurement method and composite type. Furthermore, unlike the FI method, which directly reflects fluorescence behavior under UV illumination, the SP and CP methods rely on reflected visible light, which explains the significantly higher ΔE values obtained with FI in the present study (23.01; *p* < 0.001).

According to the findings in Table 2, marked optical differences were observed between the single-shade and group-shade composites. While the single-shade composites exhibited similar, moderate ΔE_SP_ and ΔE_CP_ values, their ΔE_FI_ values were significantly higher. This finding is partially inconsistent with the results of Tüter Bayraktar et al. [14], who reported moderate ΔE_FI_ values for single-shade composites. Although both studies used similar imaging methods, these differences may be attributed to variations in the optical properties of the adhesive systems (Prime & Bond Universal vs. Clearfil Universal Bond Quick) and the spectral distributions of the light-curing units (Valo Grand vs. ZenoLite).

The markedly lower ΔE_FI_ values observed for the group-shade composite GAC indicate that this material exhibits more controlled and stable fluorescence behavior. Likewise, Tüter Bayraktar et al. [14] reported a ΔE_FI_ value of 7.66 for GAC. The Bis-MEPP-based resin matrix structure of GAC may modulate light distribution within the material, in accordance with a previous study [29] suggesting that resin composition can influence the optical properties of restorative materials. This characteristic may have contributed to the more stable fluorescence response observed in the present study. In addition, the significant difference between the SP and CP methods for this material alone suggests that its optical behavior may be more sensitive to the measurement approach. In contrast, composites containing UDMA and/or Bis-GMA (OC, CTO, VAU, OPS, and ESQ) demonstrated higher ΔE_FI_ values, suggesting that these monomer systems may affect fluorescence emission. Among these materials, OC differs from the other single-shade composites by utilizing structural color technology based on uniformly sized suprananospherical fillers rather than conventional pigment-based shade reproduction. This optical concept was primarily developed to optimize color adjustment under visible light; however, it does not necessarily ensure comparable fluorescence behavior under UV illumination, which may explain the differences observed between color adjustment and fluorescence matching in the present study. Unlike the other single-shade composites, ZC exhibited fluorescence behavior similar to that of GAC, which may be associated with its relatively lower translucency and heterogeneous filler particle structure. The multiple interfaces arising from heterogeneous particle distributions may increase light scattering and modulate the optical path length, thereby contributing to more balanced and stable fluorescence emission [29].

Although all the single-shade materials evaluated in this study belong to the same general category, they are based on different optical concepts and material compositions. OC differs from the other single-shade composites by utilizing structural color generated by uniformly sized suprananospherical fillers, whereas CTO, VAU, ZC, and OS rely on different resin matrices and filler architectures to achieve shade adaptation. Consequently, differences in resin chemistry, filler morphology, filler loading, translucency, and refractive index matching may influence light scattering and the interaction of UV light within the material, leading to material-dependent fluorescence matching.

The high ΔE_FI_ value observed for ESQ (25.14), comparable to that of the single-shade composites, indicates that the optical behavior of this material cannot be explained solely by shade-based classification. In ESQ, which incorporates suprananospherical filler technology, the homogeneous spherical particle morphology may contribute to a pronounced optical response under fluorescence conditions by enabling more controlled light distribution within the material. In contrast, the high ΔE_FI_ values observed in single-shade composites may be associated with their high translucency and reduced structurally induced environmental color adjustment under fluorescence conditions. Therefore, although similar ΔE_FI_ values were obtained in both material groups, these findings are likely attributable to different optical mechanisms. These results suggest that the fluorescence behavior of composite resins is closely related to material-specific factors, such as filler particle structure and the material’s intrinsic optical properties.

Evidence on the use of the b* parameter in the RGB color space to evaluate fluorescence properties remains limited. De Lima et al. [30] proposed an alternative approach, suggesting that changes in the b* parameter could be used to assess fluorescence behavior. Similarly, Brokos et al. [8] and Hirata et al. [9] evaluated the fluorescence properties of different composite materials using b* data from digital photographs. These approaches provide a methodological basis for comparing fluorescence measurements. Consistent with the findings of a previous study [14], the agreement between the b* parameter and fluorescence values in the present study supports its use as an indirect indicator of fluorescence. Since fluorescence emits light at longer wavelengths under UV excitation, it may have caused pronounced changes, particularly in the b* parameter, thereby increasing the overall color difference. These findings demonstrate that the optical performance of single-shade composites depends not only on intrinsic material properties but also on the evaluation method used, thereby contributing to the existing literature.

Although fluorescence matching was evaluated by calculating color differences under standardized fluorescence illumination, the intrinsic fluorescence intensity of each composite material was not directly quantified. Therefore, the present findings should be interpreted as an assessment of clinically relevant fluorescence matching rather than absolute fluorescence emission. Future studies employing calibrated fluorescence imaging systems or spectrofluorometric techniques may provide complementary quantitative information regarding the intrinsic fluorescence characteristics of restorative materials.

This study has several limitations. First, all measurements were performed immediately after specimen preparation, preventing evaluation of time-dependent changes that may affect the optical properties of the materials, such as water absorption, aging, and post-polymerization processes. In addition, the study was limited to cervical restorations; therefore, the findings may not generalize directly to restorations with different cavity geometries, depths, or material thicknesses. Furthermore, although all photographic analyses were performed under standardized imaging conditions, including identical camera settings, illumination, and background, neither colorimetric sensor calibration nor fluorescence calibration using a reference material was performed before image acquisition. Therefore, minor variations in camera-related color and fluorescence intensity cannot be entirely ruled out.

## 5. Conclusions

Within the limitations of this study, single-shade composites exhibited greater optical variability under fluorescent lighting, whereas the group-shade system demonstrated more favorable and predictable performance in both color adjustment and fluorescence stability. Simplified-shade and multi-shade composites showed intermediate optical behavior. These findings indicate that both color adjustment and optical performance under varying lighting conditions should be considered when selecting clinical materials. Future studies should evaluate the long-term optical behavior of these materials under aging conditions and across different clinical scenarios using standardized, calibrated imaging protocols, including colorimetric and fluorescence calibration, to further improve the accuracy and clinical applicability of digital color analysis.

## Figures and Tables

**Table 1:** Characteristics of the composite resins used in the study.

Abbreviation	Composite Resin	Manufacturer	Composition	Classification	Filler Ratio (wt%/vol%)	Lot Number
OC	Omnichroma	Tokuyama Dental Corp., Tokyo, Japan	UDMA, TEGDMA, supra-nano spherical fillers (SiO_2_, ZrO_2_)	Supra-nano hybrid, single-shade	79/68	101M45
CTO	Charisma Topaz One	Kulzer GmbH, Hanau, Germany	UDMA-based matrix, barium glass fillers	Nanohybrid, single-shade	81/64	N010208
VAU	Vittra APS Unique	FGM Dental Group, Joinville, SC, Brazil	UDMA-based resin matrix, nanofillers	Nanohybrid, single-shade	80/60	131223
ZC	Zenchroma	SDI Limited, Bayswater, VIC, Australia	UDMA-based matrix, nanofillers	Nanohybrid, single-shade	75/53	2024006257
OS	ONEshade	Microcopy, Kennesaw, GA, USA	Methacrylate-based resin, inorganic fillers	Nanohybrid, single-shade	77/56	2024005380
OPS	Optishade	Kerr Corp., Orange, CA, USA	Methacrylate-based resin matrix, glass fillers	Simplified shade	81/64.5	12742591
CME	Clearfil Majesty ES-2	Kuraray Noritake Dental Inc., Tokyo, Japan	Bis-GMA, hydrophobic dimethacrylate, barium glass fillers	Simplified shade	78/40	AB0159
ESQ	Estelite Sigma Quick	Tokuyama Dental Corp., Tokyo, Japan	Bis-GMA, TEGDMA, spherical silica-zirconia fillers	Supra-nano hybrid, multi-shade	82/71	281E15
GAC	G-aenial A’chord	GC Corp., Tokyo, Japan	Bis-MEPP, barium glass, silica fillers	Nanohybrid, group-shade	82/65	240918A

UDMA, urethane dimethacrylate; TEGDMA, triethylene glycol dimethacrylate; Bis-GMA, bisphenol A-glycidyl methacrylate; Bis-MEPP, 2,2-bis(4-methacryloxypolyethoxyphenyl)propane.

**Table 2:** Comparison of ΔE values according to measurement methods and composite resins.

Composite Resins	Methods			Total		Test Statistic (H)	*p* ^x^
	SP	CP	FI				
Omnichroma	3.28 ± 1.58 ^AB^	3.50 ± 1.35 ^AB^	24.16 ± 4.64 ^C^	10.32 ± 10.49 ^b^	Composites	83.749	<0.001
Charisma Topaz One	3.57 ± 0.52 ^AB^	4.39 ± 1.64 ^AB^	30.22 ± 10.92 ^C^	12.73 ± 14.11 ^b^	Methods	599.088	<0.001
Vittra APS Unique	3.36 ± 1.53 ^AB^	3.02 ± 0.95 ^AB^	25.82 ± 7.81 ^C^	10.73 ± 11.84 ^b^	Composites × Methods	140.792	<0.001
Zenchroma	4.01 ± 1.43 ^AB^	3.14 ± 1.03 ^AB^	20.08 ± 5.62 ^CD^	9.08 ± 8.65 ^b^			
ONEshade	2.86 ± 1.98 ^AB^	3.98 ± 0.88 ^AB^	21.00 ± 2.13 ^C^	9.28 ± 8.74 ^b^			
Optishade	2.55 ± 0.61 ^AB^	3.40 ± 1.29 ^AB^	25.60 ± 5.74 ^C^	10.52 ± 11.49 ^b^			
Clearfil Majesty ES-2	4.49 ± 2.20 ^AB^	3.32 ± 1.79 ^AB^	21.92 ± 4.82 ^C^	9.91 ± 9.30 ^b^			
G-aenial A’chord	2.61 ± 1.36 ^A^	3.90 ± 1.47 ^B^	13.12 ± 1.35 ^D^	6.54 ± 5.01 ^a^			
Estelite Sigma Quick	3.30 ± 1.84 ^AB^	3.34 ± 1.94 ^AB^	25.14 ± 4.34 ^C^	10.60 ± 10.99 ^b^			
Total	3.34 ± 1.52 ^a^	3.55 ± 1.35 ^a^	23.01 ± 7.04 ^b^				

^x^ Generalized estimating equations (GEEs). ^a,b^: Means sharing the same lowercase letter are not significantly different for main effects. ^A–D^: Means sharing the same uppercase letter are not significantly different for interaction effects; mean ± standard deviation.

**Table 3:** Comparison of b_ΔE_Fl_ values according to composite resins.

Composite Resins	Mean ± SD	Median (Min–Max) /Mean Ranks	Test Statistic	*p*	Effect Size (η^2^) [95% CI]
Charisma Topaz One	211.8 ± 10.57	211 (200–223)/39.8 ^a^	37.716	<0.001 ^x^	0.825 [0.104–1.377]
Clearfil Majesty ES-2	146.4 ± 11.37	145 (135–160)/11.6 ^bc^			
Estelite Sigma Quick	210 ± 10.12	214 (192–216)/39 ^ad^			
G-aenial A’chord	129.2 ± 19.2	133 (100–149)/5.4 ^b^			
Omnichroma	198.2 ± 4.55	197 (194–206)/32.6 ^acd^			
ONEshade	152.8 ± 7.69	154 (143–161)/15.2 ^abcd^			
Optishade	158.8 ± 2.59	158 (156–162)/19.9 ^abcd^			
Vittra APS Unique	187.2 ± 16.45	179 (172–210)/30.6 ^abcd^			
Zenchroma	147 ± 15.57	154 (125–163)/12.9 ^bcd^			

^x^ Kruskal–Wallis H Test. ^abcd^: Groups sharing the same lowercase letters are not significantly different. Multiple comparisons were performed using the Dunn test with Bonferroni correction; effect size [95% CI] = eta-squared (η^2^).

## Data Availability

The original contributions presented in this study are included in the article/Supplementary Materials. Further inquiries can be directed to the corresponding author.

## Supplementary Materials

The following supporting information can be downloaded at: https://www.mdpi.com/article/10.3390/polym18151819/s1, Table S1. Pairwise comparisons for the composite main effect; Table S2. Method main effect: estimated means and pairwise effect estimates; Table S3. Simple method effects within each composite material.

## Abbreviations

The following abbreviations are used in this manuscript:

SP	spectrophotometry
CP	cross-polarized photographic analysis
FI	fluorescence-illuminated photographic analysis
ΔE_SP_	spectrophotometric ΔE
ΔE_CP_	cross-polarized photographic ΔE
ΔE_FI_	fluorescence-illuminated photographic ΔE
OM	Omnichroma
CTO	Charisma Topaz One
VAU	Vittra APS Unique
ZC	Zenchroma
OS	ONEshade
OPS	Optishade
CME	Clearfil Majesty ES-2
ESQ	Estelite Sigma Quick
GAC	G-aenial A’chord
