# Supplementary material for: Color Adjustment and Fluorescence Matching of Single-Shade Composites: Spectrophotometric and Photographic Analysis Techniques
Source: Polymers (Basel). 2026 Jul 25;18(15):1819. doi: 10.3390/polym18151819 (PMC13467580; doi:10.3390/polym18151819)
Supplement: Supplementary file 1 [file polymers-18-01819-s001.zip › polymers-4381841-supplementary.pdf]

Table S1. Pairwise comparisons for the composite main effect

Comparison	SMD*
Omnichroma vs Charisma Topaz One	-0.67
Omnichroma vs Vittra APS Unique	-0.12
Omnichroma vs Zenchroma	0.34
Omnichroma vs ONEshade	0.29
Omnichroma vs Optishade™	-0.06
Omnichroma vs Clearfil Majesty™ ES-2	0.11
Omnichroma vs G-aenial Achord	1.04
Omnichroma vs Estelite Sigma Quick	-0.08
Charisma Topaz One vs Vittra APS Unique	0.55
Charisma Topaz One vs Zenchroma	1.01
Charisma Topaz One vs ONEshade	0.95
Charisma Topaz One vs Optishade™	0.61
Charisma Topaz One vs Clearfil Majesty™ ES-2	0.78
Charisma Topaz One vs G-aenial Achord	1.71
Charisma Topaz One vs Estelite Sigma Quick	0.59
Vittra APS Unique vs Zenchroma	0.46
Vittra APS Unique vs ONEshade	0.4
Vittra APS Unique vs Optishade™	0.06
Vittra APS Unique vs Clearfil Majesty™ ES-2	0.23
Vittra APS Unique vs G-aenial Achord	1.16
Vittra APS Unique vs Estelite Sigma Quick	0.04
Zenchroma vs ONEshade	-0.06
Zenchroma vs Optishade™	-0.4
Zenchroma vs Clearfil Majesty™ ES-2	-0.23
Zenchroma vs G-aenial Achord	0.7
Zenchroma vs Estelite Sigma Quick	-0.42
ONEshade vs Optishade™	-0.34
ONEshade vs Clearfil Majesty™ ES-2	-0.17
ONEshade vs G-aenial Achord	0.76
ONEshade vs Estelite Sigma Quick	-0.36
Optishade™ vs Clearfil Majesty™ ES-2	0.17
Optishade™ vs G-aenial Achord	1.1
Optishade™ vs Estelite Sigma Quick	-0.02
Clearfil Majesty™ ES-2 vs G-aenial Achord	0.93
Clearfil Majesty™ ES-2 vs Estelite Sigma Quick	-0.19
G-aenial Achord vs Estelite Sigma Quick	-1.12

* Standardized model-estimated mean differences were calculated by dividing the GEE model-estimated mean differences by the residual standard deviation derived from the model scale parameter ($\sqrt{13.127} = 3.623$). These values represent standardized model-based contrasts and should not be interpreted as conventional Cohen's d estimates.

Table S2. Method main effect: estimated means and pairwise effect estimates

Comparison	SMD*
SP vs CP	-0.06
SP vs FI	-5.43
CP vs FI	-5.37

* Standardized model-estimated mean differences were calculated by dividing the GEE model-estimated mean differences by the residual standard deviation derived from the model scale parameter (√13.127 = 3.623). These values represent standardized model-based contrasts and should not be interpreted as conventional Cohen's d estimates.

Table S3. Simple method effects within each composite material

Composite	Comparison	SMD*
Omnichroma	SP vs CP	-0.06
Omnichroma	SP vs FI	-5.76
Omnichroma	CP vs FI	-5.7
Charisma Topaz One	SP vs CP	-0.23
Charisma Topaz One	SP vs FI	-7.36
Charisma Topaz One	CP vs FI	-7.13
Vittra APS Unique	SP vs CP	0.09
Vittra APS Unique	SP vs FI	-6.2
Vittra APS Unique	CP vs FI	-6.29
Zenchroma	SP vs CP	0.24
Zenchroma	SP vs FI	-4.43
Zenchroma	CP vs FI	-4.67
ONEshade	SP vs CP	-0.31
ONEshade	SP vs FI	-5.01
ONEshade	CP vs FI	-4.7
Optishade™	SP vs CP	-0.23
Optishade™	SP vs FI	-6.36
Optishade™	CP vs FI	-6.13
Clearfil Majesty™ ES-2	SP vs CP	0.32
Clearfil Majesty™ ES-2	SP vs FI	-4.81
Clearfil Majesty™ ES-2	CP vs FI	-5.13
G-aenial Achord	SP vs CP	-0.36
G-aenial Achord	SP vs FI	-2.9
G-aenial Achord	CP vs FI	-2.55
Estelite Sigma Quick	SP vs CP	-0.01
Estelite Sigma Quick	SP vs FI	-6.03
Estelite Sigma Quick	CP vs FI	-6.02

* Standardized model-estimated mean differences were calculated by dividing the GEE model-estimated mean differences by the residual standard deviation derived from the model scale parameter (√13.127 = 3.623). These values represent standardized model-based contrasts and should not be interpreted as conventional Cohen's d estimates.
